# Is vitamin D a modifiable risk factor for dental caries?

**DOI:** 10.12688/wellcomeopenres.16369.1

**Published:** 2020-12-01

**Authors:** Serena A. Dodhia, Nicola X. West, Steven J. Thomas, Nicholas J. Timpson, Ingegerd Johansson, Pernilla Lif Holgerson, Tom Dudding, Simon Haworth

**Affiliations:** 1 Bristol Dental School, University of Bristol, Bristol, BS1 2LY, UK; 2 Medical Research Council Integrative Epidemiology Unit, Population Health Sciences, University of Bristol, Bristol, BS8 2BN, UK; 3 Avon Longitudinal Study of Parents and Children, University of Bristol, Bristol, UK; 4 Department of Odontology, Section of Cariology, Umea University, Umeå, Sweden; 5 Department of Odontology, Section of Pedodontics, Umea University, Umeå, Sweden

**Keywords:** Vitamin D, Dental caries, Mendelian randomization, 25-hydroxyvitamin D

## Abstract

**Background:** Prior observational studies have reported that higher levels of vitamin D are associated with decreased caries risk in children. However, these studies are prone to bias and confounding so do not provide causal inference. Genetic variants associated with a risk factor of interest can be used as proxies, in a Mendelian randomization (MR) analysis, to test for causal association with an outcome. The objective was to estimate the causal association between serum 25-hydroxyvitamin D (25(OH)D) (the commonly measured vitamin D metabolite in blood) and dental caries using a MR approach which estimates the causal effect of an exposure on an outcome.

**Methods: **A total of 79 genetic variants reliably associated with 25(OH)D were identified from genome-wide association studies and used as a proxy measure of 25(OH)D. The association of this proxy measure with three outcome measures was tested; specifically: caries in primary teeth (n=17,035, aged 3-12 years), caries in permanent teeth in childhood and adolescence (n=13,386, aged 6-18 years), and caries severity in adulthood proxied by decayed, missing and filled tooth surfaces (DMFS) counts (n=26,792, aged 18-93 years).

**Results: **The estimated causal effect of a one standard deviation increase in natural log-transformed 25(OH)D could be summarized as an odds ratio of 1.06 (95%CI: 0.81, 1.31; P=0.66) for caries in primary teeth and 1.00 (95%CI: 0.76, 1.23; P=0.97) for caries in permanent teeth in childhood and adolescence. In adults, the estimated casual effect of a one standard deviation increase in natural log-transformed 25(OH)D was 0.31 fewer affected tooth surfaces (95%CI: from 1.81 fewer DMFS to 1.19 more DMFS; P=0.68)

**Conclusions: **The MR-derived effect estimates for these three measures are small in magnitude with wide confidence intervals and do not provide evidence against the null hypothesis of no effect.

## Introduction

Dental caries is a disease process which can lead to irreversible damage to tooth tissues. Initially hydroxyapatite crystals in the enamel, dentine and cementum tissues are demineralized when acidic by-products from bacterial fermentation of simple carbohydrates lead to low pH and mineral undersaturation in the tooth surrounding fluids. Eventually demineralization is followed by a proteolytic destruction of the organic substances of the tooth tissues and a cavity is formed ( Selwitz *et al*., 2007).

Both genetic and environmental risk factors influence dental caries and there is a need to identify modifiable risk factors which could be targets for effective interventions. Vitamin D has been suggested as a potential modifiable risk factor. There is an inverse association between serum 25-hydroxyvitamin D (25(OH)D) (the commonly measured vitamin D metabolite in blood) and caries in childhood ( Kim *et al*., 2018; Schroth *et al*., 2016), and potential mechanisms, including tooth mineralization and antibacterial effects, have been suggested. Vitamin D stimulates absorption of calcium ( Veldurthy *et al*., 2016), and phosphate ( Fukumoto, 2014), so may be relevant to hydroxyapatite crystal structure and mineralization. Vitamin D induces genomic effects in odontoblasts (dentine formation) and ameloblasts (enamel formation) through vitamin D receptor signalling ( Zhang *et al*., 2008). Therefore, vitamin D deficiencies during tooth formation may cause the hard tissues of the tooth to be more sensitive to demineralization. Vitamin D stimulates the production of antimicrobial peptides, such as cathelicidins, which are effective against opportunistic gram-positive and gram-negative bacteria in the tooth biofilm ( Youssef *et al*., 2011). In addition, insufficient levels of vitamin D have been reported to be associated with atrophy of the salivary glands with impaired saliva secretion and increased caries risk ( Scardina & Messina, 2012).

These studies do not allow for direct causal inference but have led to the hypothesis that vitamin D supplementation in childhood may be a way to prevent dental caries at a population level. To test this hypothesis, controlled trials have been conducted. A meta-analysis of controlled clinical trials supported causal effects of vitamin D, but also highlighted substantial heterogeneity in estimates, risk of bias in individual studies and strong evidence for publication bias in the available literature ( Hujoel, 2013). There is therefore a need for additional sources of evidence to help clarify the causal effect of vitamin D in dental caries.

Mendelian randomization (MR) is an alternative way to estimate causal effects, when randomized controlled trials are unfeasible or inconclusive. This method uses genetic variation, which is reliably associated with the exposure of interest, as a proxy measure of the exposure. These genetic variations are often single base pair changes in germline genotypes, termed single nucleotide polymorphisms or SNPs. A higher number of 25(OH)D increasing variants is associated with higher average serum 25(OH)D concentration. Due to the essentially random assortment of alleles during meiosis, the proxy measure of 25(OH)D concentration is unrelated to traditional confounding factors at population level. For example, individuals with two 25(OH)D increasing alleles at a particular SNP will smoke no more or less than those who have zero 25(OH)D increasing alleles at the same SNP. This is in contrast to serum 25(OH)D measures which, at a population level, show a strong inverse association with smoking ( Kassi *et al*., 2015).

In addition, since disease processes (caries in this case) cannot alter germline genotypes, the direction of causation is from the proxy measure of exposure to the outcome, therefore reducing the risk of bias from reverse causation ( Lawlor *et al*., 2008). MR has been increasingly used over the past decade, to provide more robust causal effect estimates for a range of risk factors and health outcomes ( Davies *et al*., 2018).

A previous study has assessed the causal effect of 25(OH)D on caries using MR, but was underpowered to provide precise estimates or fully interrogate the assumptions of the MR method ( Dudding *et al*., 2015). Since then, developments in methodology ( Bowden *et al*., 2015; Bowden *et al*., 2016) and the understanding of vitamin D genetics ( Jiang *et al*., 2018; Manousaki *et al*., 2017; Manousaki *et al*., 2020) has resulted in an increase in the strength of its genetic proxy. There have also been larger studies with dental caries traits and genetic data published ( Haworth *et al*., 2018; Shungin *et al*., 2019). These developments create the opportunity to re-examine the association of 25(OH)D and dental caries.

The objective of this study is to use MR to assess the causal role of serum 25(OH)D on three caries related traits (caries in the primary dentition, caries in the permanent dentition in children and adolescents, and caries severity in adults) using data from published genome-wide association studies (GWASs).

## Methods

### SNP-exposure: identifying SNPs as 25(OH)D proxies

SNPs to be used as proxies for 25(OH)D exposure were identified as those that a) were strongly associated with 25(OH)D exposure (defined as P≤5×10 ^-8^ in at least one published GWAS); b) were identified in a population of European ancestry participants; c) were not in the same genomic region as another 25(OH)D associated SNP, unless there was published evidence showing that SNPs had independent effects and d) had a minor allele frequency of 0.05 (5%) or above in the 25(OH)D GWAS and the caries outcome GWASs. For each variant, information about the strength and direction of association with 25(OH)D was extracted and recorded alongside other information such as variant name and nearest gene.

### SNP-outcome: obtaining estimates of the association between 25(OH)D–proxying SNPs and caries outcomes

GWASs test for association between millions of SNPs across the genome and diseases such as dental caries. Existing GWAS studies can therefore be used to obtain estimates of the association between genetic proxies for an exposure of interest and an outcome. For this investigation, the estimated effects of 25(OH)D–proxying SNPs on dental caries were obtained by extracting association statistics for these variants from published results.

SNP-caries association estimates for caries in the primary dentition in children and caries in the permanent dentition in adolescents were obtained from a published meta-analysis. This meta-analysis was originally carried out in a consortium, including cohort studies in the USA, UK, Denmark, Finland, the Netherlands, Germany and Australia ( Haworth *et al*., 2018). Each of these studies classified participants as caries-free or caries-affected using clinical examination, index linkage to pre-existing dental records or using intra-oral photographs. One contributing study used child- and parent-reported questionnaires to classify children as caries-free or caries-affected. The relationship between GWAS derived SNPs and caries status was estimated in a logistic regression framework accounting for co-variables such as age, sex and genetic principal components, which aim to control for variation in genetic data which is due to genetic ancestry. The log-odds ratio (OR) effect estimates were combined in a fixed-effects genome-wide meta-analysis. The principal analysis included all studies with phenotypic data, and a sensitivity analysis was performed which excluded participants with questionnaire-derived caries status. Results from the principal meta-analysis were download from the University of Bristol research data repository and are available at https://doi.org/10.5523/bris.pkqcnil6e9ju2nyreblt3mvwf ( Haworth & Timpson, 2018). Downladed data were used to extract the SNP-outcome information about the vitamin D-associated SNPs, for caries in the primary and caries in the permanent dentition in children and adolescents, respectively.

SNP-caries association estimates for decayed, missing and filled tooth surfaces (DMFS) were obtained from a genome-wide meta analysis of adult cohort studies based in the USA, Germany, Sweden and Finland ( Shungin *et al*., 2019). In each study, DMFS scores were calculated excluding third molar teeth and root caries, and were derived from surface-level dental charts, which were either obtained as part of the study protocol or obtained via index linkage to pre-existing dental records. Within each study, DMFS scores were residualized on covariates including age, age squared and sex, then residuals were standardized to a mean of 0 and standard deviation of 1. Subsequently, the relationship between SNPs and transformed DMFS scores was estimated using a linear regression framework, and beta coefficients were combined across studies using a genome-wide fixed-effects meta-analysis. The principal analysis included all studies, while a sensitivity analysis was performed excluding one study of Hispanic/Latino participants. Results of the primary and sensitivity meta-analyses were downloaded from the University of Bristol research data repository at https://doi.org/10.5523/bris.2j2rqgzedxlq02oqbb4vmycnc2 ( Haworth, 2019). This dataset was used to extract the SNP-outcome information about the 25(OH)D-associated SNPs for the caries severity in adults outcome.

### Causal effect estimation

Intuitively, if SNPs which are proxies for 25(OH)D are associated with caries outcomes then there must be an effect of 25(OH)D on caries. This intuition is formalized using a series of statistical tests under an analytical paradigm termed two-sample MR ( Burgess *et al*., 2015). In this investigation, the “ TwoSampleMR” package version 0.5.4 in R version 3.4.3 ( Hemani *et al*., 2016) was used. For the primary analysis, causal effect estimates were obtained separately for each SNP and then combined using inverse variance weighted (IVW) meta-analyses to estimate the overall causal effect of serum 25(OH)D on each of the three outcomes.

For caries in the primary dentition and permanent dentition in children and adolescents each causal effect estimate (β *_xy_*) and standard error (SE) from the two-sample MR was converted into an interpretable OR with 95% confidence intervals (CIs) as follows:

$$\text{Causal}\;\text{OR}\;={\;}_{e}\left({\text{β}}_{xy}\right)$$

$$95\%\;\text{CIs}\;={\;}_{e}\left({\text{β}}_{xy}\pm \;\left(1.96\;\times \;\text{SE}\right)\right)$$

As the causal effect estimates for DMFS are not in meaningful units, the β _*xy*_ and accompanying CIs were back transformed to give number of affected tooth surfaces. As reference, 19.87 DMFS corresponded to a 1-unit change in the transformed DMFS score in a population of 28,691 adults (aged 30–75 years) in the Swedish GLIDE database who were originally recruited through the Northern Sweden Health and Disease Study ( Hallmans *et al*., 2003). This value was used to transform the causal effect and corresponding CIs from a standardized scale to a tooth surface scale with 95% CIs as follows:

$$\text{Causally}\;\text{affected}\;\text{tooth}\;\text{surfaces}\;\left(\text{DMFS}\right)\;\text{=}\;{\text{β}}_{xy}\;\times \;19.87$$

$$\text{95\%}\;\text{CIs}\;\text{=}\;\text{19.87}\;\left({\text{β}}_{xy}\;\pm \;\left(\text{1.96}\;\text{×}\;\text{SE}\right)\right)$$

*Leave-one-out sensitivity analysis*

For each outcome, a leave-one-out sensitivity analysis was undertaken. In this analysis, the IVW analysis was carried out iteratively omitting one 25(OH)D proxying SNP in turn. This analysis aims to identify SNPs with outlying causal effect estimates and ensures that the overall estimate of the causal effect of 25(OH)D on dental caries was not driven by a single or few SNPs with large effects. In cases where a small number of SNPs have large effects on caries (but not on 25(OH)D), this may imply that these SNPs act on caries through mechanisms unrelated to vitamin D (pleiotropic effects) and violate the core assumptions of the MR method.

*Weighted median sensitivity analysis*

For each outcome, a weighted median (WM) sensitivity analysis of the SNPs was undertaken. The WM provides a consistent estimate of the causal effect, provided that 50% of the weight in the analysis stems from non-pleiotropic, and therefore valid, variants.

*MR-Egger regression sensitivity analysis*

For each outcome, a MR-Egger regression of the SNP-outcome on the SNP-exposure with the y-intercept unconstrained was carried out. The y-intercept tests for the presence of directional pleiotropy, since when the SNP-exposure association is zero the SNP-outcome association should also be zero. The MR-Egger regressions rely on the instrument strength independent of direct effect (InSIDE) assumption that the strength of the SNP-exposure association should not correlate with the strength of any pleiotropic effects across the instrumental variants ( Bowden *et al*., 2015).

*Strength of instrumental variables*

Instrumental variable (IV) estimates can suffer from weak instrument bias, which arises when confounders in the genotypic subgroups in samples are not perfectly balanced. If the IVs are weak, they explain less variation in the phenotype and therefore the difference in confounders between the subgroups might explain more of the variation in phenotype. If this bias is present, a confounded false-positive association between exposure and outcome may be found ( Burgess *et al*., 2011). The F-statistic from a regression of 25(OH)D on the proxy SNPs provides an indication of the reliability of the correlation between instrument and intermediate ( Burgess *et al*., 2011). F-statistics were generated for each outcome using a free web-based MR F-statistic calculator ( http://cnsgenomics.com/shiny/mRnd/) to provide some comment on the strength of the genetic instruments in each analysis.

*Power*

Post-hoc power calculations were carried out for each outcome to assess the causal effect estimate sizes that this study was powered to detect. The total variance explained of the 25(OH)D proxy measure was calculated by summing the variance explained for each independent SNP using the formula: variance explained ≈ 2 *β* ^2^ƒ(1 – ƒ), where β and ƒ denote the effect estimate and the effect allele frequency of the allele on a standardized phenotype, respectively ( Park *et al*., 2010). A web-based MR power calculator was used ( http://cnsgenomics.com/shiny/mRnd/).

### Ethical approval

This analysis of previously published data was conducted in accordance with principles described in the Helsinki declaration and all addition requirements within the United Kingdom. All participants participating in the published studies which contributed to this analysis gave informed consent, as described in the respective publications.

## Results

### SNP-exposure: identifying SNPs as 25(OH)D proxies

In total, 83 SNPs met the criteria for inclusion as proxies for 25(OH)D; these were selected from the most recent published GWAS of 25(OH)D ( Manousaki *et al*., 2020). However, seven SNPs associated with 25(OH)D identified from the SNP-exposure stage were not present in the outcome data for all three traits and one SNP (rs200454003) was not present in the outcome data for caries in primary teeth and caries in permanent teeth in paediatric populations. Proxy SNPs with the highest linkage disequilibrium in European populations (r ^2^) to the seven missing SNPs were identified. Proxy SNPs were used for four SNPs (rs2934744, rs7650253, rs3822868, rs201501563) where the r ^2^ was 0.7 or greater. The remaining three missing SNPs (rs145432346, rs200641845, rs3775150) were excluded from the exposure data as no suitable proxy was identified, leaving 79 SNPs for analysis for caries in primary teeth and caries in permanent teeth in paediatric populations.

For DMFS, two SNPs (rs10127775 and rs10832289) were removed for being palindromic with intermediate allele frequencies (to harmonize the data so that the effect of the variants both exposure and outcome corresponded to the same allele), leaving 78 SNPs for analysis. Information about the 79 SNPs is provided in Table 1. In total, these SNPs explain approximately 4.4% of the total variation in 25(OH)D levels in populations of European ancestry.

**Table 1.** Summary of the Vitamin D SNPs extracted for analysis.

SNP	Chromosome	Position ^†^	Nearest Gene	Allele 1	Allele 2	Allele 1 Frequency	Beta	SE	P value ^*^
rs6698680	1	2329661	RER1	G	**A**	0.464	-0.012	0.002	8.99E-10
rs3750296	1	17559656	*PADI1*	C	G	0.341	-0.021	0.002	2.09E-24
rs7519574	1	34726552	*RP4-657M3.2*	A	G	0.182	0.017	0.003	2.09E-11
rs56044892	1	41830086	*FOXO6*	T	C	0.211	0.015	0.002	2.85E-10
rs2934744	1	63048045	*DOCK7*	A	C	0.644	-0.022	0.002	3.96E-26
rs7528419	1	109817192	*CELSR2*	G	A	0.225	0.019	0.002	2.41E-16
rs3768013	1	150815411	*ARNT*	A	G	0.370	-0.015	0.002	1.37E-13
rs11264360	1	155284586	*FDPS*	A	T	0.243	0.018	0.002	3.34E-15
rs867772	1	220972343	*MARC_1*	G	A	0.682	-0.014	0.002	3.64E-11
rs10127775	1	230295789	*GALNT2*	T	A	0.605	0.012	0.002	3.43E-09
rs12997242	2	21381177	*TDRD15*	A	G	0.438	-0.013	0.002	2.23E-10
rs11127048	2	27752463	*GCKR*	A	G	0.617	0.018	0.002	6.41E-19
rs6724965	2	101440151	*NPAS2*	G	A	0.172	-0.017	0.003	1.29E-10
rs7569755	2	118648261	*HTR5BP*	A	G	0.292	0.014	0.002	8.03E-11
rs1047891	2	211540507	*CPS1*	A	C	0.316	-0.014	0.002	1.16E-11
rs2011425	2	234627608	*UGT1A4*	G	T	0.079	-0.046	0.004	9.66E-38
rs7650253	3	49431160	*RHOA*	A	T	0.690	0.015	0.002	1.76E-10
rs1972994	3	85631142	*CADM2*	T	A	0.647	-0.018	0.002	7.99E-18
rs6438900	3	125148287	*MRPL3*	G	C	0.261	0.014	0.002	9.59E-10
rs6773343	3	141825598	*TFDP2*	T	C	0.720	0.013	0.002	5.20E-09
rs78649910	4	3482213	*DOK7*	A	T	0.110	-0.018	0.003	4.32E-09
rs7699711	4	69947596	*UGT2B7*	T	G	0.455	-0.029	0.002	6.97E-49
rs145432346	4	72575017	*GC*	C	T	0.826	0.109	0.003	6.78E-286
rs705117	4	72608115	*GC*	T	C	0.849	-0.034	0.003	1.71E-36
rs11723621	4	72615362	*GC*	G	A	0.291	-0.187	0.002	2.903E-1689
rs200641845	4	72620895	*GC*	T	A	0.545	0.018	0.002	6.92E-14
rs3775150	4	72640750	*GC*	C	T	0.262	-0.091	0.002	3.90E-295
rs222026	4	72643760	*GC*	T	A	0.871	-0.052	0.003	6.98E-68
rs186881826	4	72785743	*GC*	A	T	0.223	0.046	0.002	3.64E-77
rs58073039	4	88287363	*HSD17B11*	G	A	0.298	-0.014	0.002	2.16E-11
rs7718395	5	118652574	*TNFAIP8*	G	C	0.320	0.013	0.002	1.67E-09
rs3822868	6	131934986	*MED23*	G	A	0.835	0.022	0.003	1.41E-15
rs111529171	7	21571932	*DNAH11*	C	G	0.216	-0.015	0.002	6.24E-11
rs1011468	7	104613791	*LINC01004*	A	G	0.476	-0.014	0.002	1.35E-12
rs1858889	7	107117447	*COG5*	C	A	0.501	0.013	0.002	3.85E-11
rs804280	8	11612698	*GATA4*	A	C	0.582	0.013	0.002	4.43E-11
rs34726834	8	25889606	*EBF2*	T	C	0.254	0.014	0.002	6.65E-10
rs7828742	8	116960729	*LINC00536*	G	A	0.597	-0.022	0.002	3.06E-28
rs10818769	9	125719923	*DNAH11*	G	C	0.857	-0.017	0.003	3.35E-09
rs532436	9	136149830	*ABO*	A	G	0.184	-0.015	0.003	2.17E-09
rs10887718	10	82042624	*MAT1A*	T	C	0.527	-0.012	0.002	1.44E-10
rs10832218	11	14181174	*CYP2R1*	C	T	0.198	-0.034	0.003	7.09E-32
rs10832289	11	14669496	*CYP2R1*	T	A	0.410	-0.069	0.002	2.03E-266
rs201501563	11	14882470	*CYP2R1*	T	C	0.122	-0.066	0.004	9.17E-67
rs523583	11	66070146	*TMEM151A*	C	A	0.469	0.012	0.002	5.58E-10
rs12803256	11	71132868	*FLJ42102*	G	A	0.771	0.100	0.002	8.599E-407
rs200454003	11	71228990	*FLJ42102*	T	C	0.265	-0.087	0.003	3.68E-256
rs10793129	11	75459865	*RP11-21L23.4*	A	G	0.090	0.024	0.003	1.64E-12
rs1149605	11	76485216	*RP11-21L23.4*	C	T	0.171	0.019	0.003	7.34E-14
rs964184	11	116648917	*ZPR1*	C	G	0.864	0.040	0.003	5.11E-44
rs2847500	11	120114421	*ZPR1*	A	G	0.124	-0.021	0.003	7.79E-13
rs12317268	12	21352541	*SLCO1B1*	G	A	0.152	-0.019	0.003	9.15E-12
rs9668081	12	38602911	*FAM166AP9*	T	C	0.471	0.012	0.002	5.38E-09
rs10859995	12	96375682	*HAL*	C	T	0.581	-0.039	0.002	7.03E-89
rs8018720	14	39556185	*SEC23A*	C	G	0.820	-0.032	0.003	4.04E-36
rs261291	15	58680178	*LIPC*	C	T	0.356	-0.022	0.002	2.89E-28
rs1800588	15	58723675	*LIPC*	T	C	0.215	-0.030	0.002	2.65E-36
rs17765311	15	63789952	*AC007950.2*	C	A	0.345	-0.015	0.002	1.35E-13
rs62007299	15	77711719	*PEAK1*	A	G	0.709	-0.014	0.002	1.69E-11
rs8063706	16	11909552	*BCAR4*	T	A	0.273	0.013	0.002	3.64E-09
rs77924615	16	20392332	*PDILT*	A	G	0.198	-0.016	0.002	1.46E-10
rs71383766	16	30930233	*FBXL19*	T	C	0.420	0.013	0.002	1.15E-09
rs1800775	16	56995236	*CETP*	A	C	0.486	-0.017	0.002	1.56E-17
rs2909218	17	66464546	*RP11-120M18.2*	T	C	0.793	0.017	0.002	2.81E-12
rs8091117	18	28919794	*DSG1*	A	C	0.065	-0.024	0.004	1.03E-09
rs2037511	18	61366207	*SERPINB11*	A	G	0.165	0.016	0.003	9.29E-10
rs57631352	19	4338173	*STAP2*	G	A	0.297	-0.013	0.002	1.48E-09
rs73015021	19	11192915	*LDLR*	G	A	0.121	0.023	0.003	1.15E-14
rs10500209	19	11979164	*LDLR*	C	T	0.282	-0.013	0.002	6.18E-10
rs58542926	19	19379549	*TM6SF2*	T	C	0.076	0.032	0.004	8.57E-19
rs3814995	19	36342212	*NPHS1*	T	C	0.312	-0.015	0.002	2.83E-12
rs1065853	19	45413233	*APOC1*	T	G	0.082	0.027	0.004	8.32E-14
rs157595	19	45425460	*APOC1*	G	A	0.614	-0.016	0.002	2.95E-14
rs112285002	19	48374320	*SULT2A1*	T	C	0.160	0.060	0.003	1.77E-110
rs62130059	19	48461240	*SULT2A1*	C	A	0.336	-0.027	0.002	9.25E-34
rs10426	19	51517798	*KLK10*	A	G	0.213	0.025	0.002	3.31E-26
rs8103262	19	53065814	*ZNF808*	C	T	0.305	0.013	0.002	3.18E-09
rs6123359	20	52714706	*RP13-379L11.3*	G	A	0.105	0.032	0.003	7.74E-24
rs6127099	20	52731402	*RP13-379L11.3*	T	A	0.279	-0.037	0.002	9.30E-62
rs2585442	20	52737123	*RP13-379L11.3*	G	C	0.246	0.034	0.002	6.87E-49
rs2229742	21	16339172	*NRIP1*	C	G	0.104	-0.026	0.003	7.13E-16
rs2074735	22	31535872	*PLA2G3*	C	G	0.064	0.027	0.004	6.55E-12
rs960596	22	41393520	*SCUBE1*	T	C	0.340	0.012	0.002	2.23E-09

SNP: single nucleotide polymorphism; SE: standard error. *P value tests the null hypothesis of no association with 25(OH)D ^†^Positions are reported according to Genome Reference Consortium Human Build 37 (GRCh37/hg19).

### SNP-outcome: obtaining estimates of the association between 25(OH)D–proxying SNPs and caries outcomes

For caries in primary teeth, binary data for 17,035 children (aged 3–12 years) were available from nine studies of European ancestry children. Overall, 41% of participants were classified as having caries (6,922 caries-affected, 10,113 caries-free). For caries in the permanent dentition in children and adolescents, binary data on 13,386 participants (aged 6–18 years) were available from seven studies of European ancestry. In total, 44% were classified as having caries (5,875 caries-affected, 7,511 caries-free). For DMFS, quantitative data on 26,792 adults (aged 18–93 years) were available from nine studies (eight studies were of European ancestry and one study was of admixed Hispanic/Latino ancestry). For each of these studies, SNP-caries effect estimate summary statistics were extracted for the 81 SNPs associated with 25(OH)D ( Table 2).

**Table 2.** Summary of SNP-outcome association statistics for the three caries outcomes.

			Caries in Primary teeth					Caries in Permanent teeth					DMFS				
SNP	A1	A2	A1F	Beta	SE	P	N	A1F	Beta	SE	P	N	A1F	Beta	SE	P	N
rs7528419	a	g	0.7715	-0.0398	0.0356	0.2641	16586	0.7784	0.029	0.0369	0.4311	12937	0.7889	0.0078	0.0106	0.4625	26790
rs3768013	a	g	0.3611	0.029	0.0306	0.3439	17029	0.3645	0.06	0.0315	0.05649	13382	0.426	-0.0074	0.009	0.4108	26790
rs11264360	a	t	0.2455	0.0043	0.0345	0.8997	17023	0.2454	0.0359	0.0356	0.3139	13383	0.3044	-0.0103	0.0102	0.3107	26791
rs3750296	c	g	0.3472	-0.0313	0.0305	0.3044	17032	0.3503	-0.0269	0.0312	0.3879	13384	0.3728	0.0133	0.0093	0.1507	26789
rs867772	a	g	0.3059	-0.0212	0.0325	0.5129	16945	0.3112	-0.0437	0.0328	0.183	13359	0.3268	0.0012	0.01	0.9073	26792
rs10127775	a	t	0.4057	0.0107	0.0308	0.7277	16520	0.4003	0.0494	0.0311	0.112	12927	0.4673	-0.0008	0.0088	0.9285	26791
rs6698680	a	g	0.5311	0.0085	0.0297	0.775	17022	0.5344	-0.0036	0.0306	0.907	13383	0.6092	-0.0017	0.0088	0.844	26790
rs7519574	a	g	0.1692	-0.0617	0.0486	0.204	11767	0.1671	-0.0232	0.0447	0.6033	7999	0.1836	0.0096	0.0127	0.4483	26791
rs56044892	t	c	0.2118	0.021	0.0375	0.5761	16926	0.213	0.0012	0.0379	0.9742	13347	0.22	0.0072	0.0129	0.5796	26789
rs10887718	t	c	0.5215	-0.0332	0.0291	0.2543	17037	0.5247	-0.0317	0.0299	0.2902	13386	0.6486	-0.002	0.0089	0.8233	26790
rs964184	c	g	0.8614	0.1134	0.0538	0.03506	11318	0.8602	0.052	0.0489	0.2876	7551	0.8952	-0.0167	0.011	0.1274	26789
rs2847500	a	g	0.1255	-0.0796	0.0462	0.0845	16952	0.1232	0.0182	0.0481	0.7058	13351	0.2265	0.036	0.0121	0.002913	26791
rs10832218	t	c	0.6948	-0.0223	0.0388	0.566	17037	0.74	-0.0027	0.0435	0.9509	13386	0.6556	0.0117	0.0089	0.1927	26791
rs10832289	a	t	0.5866	0.0332	0.0298	0.2653	17037	0.5874	0.0193	0.0305	0.5266	13386	0.628	0.0161	0.0088	0.06729	26791
rs523583	a	c	0.529	-0.0026	0.0358	0.9424	11767	0.5257	-0.0254	0.0332	0.4453	7999	0.5697	-0.0104	0.0088	0.2338	26791
rs12803256	a	g	0.2959	0.0167	0.0325	0.6076	17018	0.2741	0.0204	0.0343	0.5516	13378	0.5016	0.0161	0.009	0.07419	26792
rs10793129	a	g	0.1022	0.0191	0.0875	0.8272	4707	0.0949	0.0029	0.1207	0.981	2079	0.1507	0.0316	0.0146	0.03075	26790
rs1149605	t	c	0.8303	-0.0663	0.0392	0.09072	17037	0.8273	-0.1035	0.04	0.009713	13386	0.8776	-0.0167	0.0124	0.1767	26792
rs12317268	a	g	0.8293	0.0422	0.0482	0.3808	11767	0.8355	-0.0478	0.045	0.2884	7999	0.8515	-0.015	0.0116	0.1983	26790
rs9668081	t	c	0.4807	-0.0715	0.0527	0.1749	4679	0.4758	0.0012	0.069	0.9865	2071	0.5563	-0.006	0.0087	0.4886	26791
rs10859995	t	c	0.4209	0.0549	0.0357	0.1244	11767	0.4228	-0.0052	0.0333	0.8761	7999	0.5772	-0.0099	0.0088	0.2578	26791
rs8018720	c	g	0.8336	-0.0313	0.0404	0.4384	16585	0.8273	-0.0808	0.0413	0.0503	12937	0.8762	0.0229	0.0113	0.04263	26791
rs261291	t	c	0.6454	-0.0072	0.0305	0.8145	17027	0.6439	0.0217	0.0315	0.4915	13379	0.6703	0.0022	0.009	0.8094	26790
rs1800588	t	c	0.2144	-0.0273	0.0367	0.4563	16582	0.2113	0.0153	0.0381	0.687	12938	0.4643	-0.007	0.0095	0.4646	26790
rs17765311	a	c	0.646	-0.0421	0.0314	0.1797	16556	0.6493	0.012	0.0324	0.7107	12924	0.7866	-0.0167	0.0097	0.08395	26790
rs62007299	a	g	0.7111	0.0149	0.0321	0.6416	17035	0.7134	0.0339	0.033	0.3052	13385	0.741	-0.004	0.0093	0.6685	26790
rs8063706	a	t	0.7121	0.0664	0.0342	0.05258	16344	0.7204	0.0659	0.0339	0.05223	13350	0.7396	0.0002	0.01	0.9868	26790
rs77924615	a	g	0.2002	0.0277	0.0377	0.4623	16966	0.2008	-0.0209	0.0383	0.5846	13361	0.2262	0.0069	0.011	0.5283	26789
rs71383766	t	c	0.3967	-0.0534	0.0611	0.3818	4579	0.4139	-0.0694	0.0786	0.377	1865	0.4351	-0.0071	0.0093	0.4419	26790
rs1800775	a	c	0.4822	-0.0302	0.0369	0.412	11318	0.4855	-0.0543	0.0339	0.1088	7550	0.5197	0.0033	0.0087	0.708	26789
rs2909218	t	c	0.7987	0.0284	0.0365	0.4361	17025	0.7992	0.0058	0.0378	0.8787	13384	0.8359	-0.0022	0.0101	0.8276	26791
rs8091117	a	c	0.0721	0.1079	0.0716	0.1317	11318	0.0674	0.1266	0.0678	0.06196	7551	0.149	0.0106	0.014	0.4469	26791
rs2037511	a	g	0.1653	0.0182	0.0494	0.7126	11318	0.1623	0.032	0.0459	0.4863	7551	0.1939	0.0054	0.0117	0.6434	26790
rs73015021	a	g	0.8841	0.0861	0.0466	0.06437	17024	0.8803	0.0302	0.0471	0.5211	13386	0.9088	0.0293	0.013	0.02379	26792
rs10500209	t	c	0.7193	-0.0767	0.0403	0.05718	11317	0.7215	-0.056	0.0373	0.1337	7551	0.8095	0.0098	0.0102	0.336	26791
rs58542926	t	c	0.0813	0.0191	0.056	0.7332	16584	0.0816	-0.1306	0.0566	0.02103	12938	0.109	0.0062	0.017	0.7127	26791
rs3814995	t	c	0.3177	-0.0489	0.0403	0.2248	13471	0.3164	-0.0195	0.0375	0.6037	12724	0.3704	0.0069	0.0099	0.4851	26791
rs57631352	a	g	0.7039	-0.0281	0.0318	0.3775	17032	0.7062	-0.0158	0.0332	0.6337	13383	0.7205	-0.0121	0.0095	0.2048	26790
rs1065853	t	g	0.0745	0.0724	0.2229	0.7453	644	0.0693	-0.2843	0.5623	0.6131	202	0.0786	0.1254	0.0795	0.1146	1116
rs157595	a	g	0.3653	-0.0109	0.0338	0.747	15328	0.3711	-0.0409	0.0332	0.2176	13172	0.5123	-0.0003	0.0095	0.9767	26791
rs112285002	t	c	0.1073	-0.2394	0.0987	0.01527	4608	0.12	-0.0908	0.119	0.4452	2051	0.1274	0.0092	0.0185	0.6194	26791
rs62130059	a	c	0.4583	-0.0481	0.0969	0.6197	1591	0.4537	-0.0174	0.09	0.8464	1865	0.6457	0.0115	0.0103	0.2643	26791
rs10426	a	g	0.2184	0.0879	0.0438	0.04488	11766	0.2207	0.0615	0.0405	0.1292	7999	0.2535	-0.0141	0.0114	0.2175	26790
rs8103262	t	c	0.6948	-0.0274	0.0317	0.388	17030	0.6963	0.0025	0.033	0.9406	13386	0.7214	0.0115	0.0093	0.2167	26790
rs6724965	a	g	0.8233	-0.0312	0.0384	0.4174	17014	0.8248	0.0626	0.0394	0.112	13381	0.8299	0.0292	0.0103	0.004574	26792
rs7569755	a	g	0.2819	0.0107	0.0322	0.7387	17032	0.285	0.0478	0.0331	0.1491	13384	0.2943	-0.0006	0.0101	0.9555	26791
rs1047891	a	c	0.318	-0.0028	0.0346	0.9356	15895	0.3208	0.0051	0.0343	0.8819	12724	0.3562	0.0191	0.0095	0.04407	26791
rs12997242	a	g	0.4302	-0.0229	0.03	0.4461	16588	0.4346	-0.0295	0.0309	0.3392	12938	0.4487	0.0027	0.0089	0.7603	26791
rs2011425	t	g	0.9174	-0.0563	0.0539	0.2958	17034	0.9169	-0.0554	0.0556	0.3198	13386	0.937	-0.0044	0.015	0.7695	26791
rs11127048	a	g	0.5689	-0.0464	0.0565	0.412	4674	0.5503	-0.0883	0.0735	0.2298	2070	0.6373	0.02	0.0092	0.02982	26790
rs6123359	a	g	0.901	-0.1575	0.0511	0.002053	17024	0.8982	-0.0575	0.0504	0.2544	13383	0.9227	-0.0161	0.014	0.2512	26789
rs6127099	a	t	0.7272	0.0092	0.035	0.7932	16344	0.7268	-0.003	0.0347	0.9303	13172	0.764	-0.0011	0.0095	0.9087	26789
rs2585442	c	g	0.7451	-0.0194	0.0343	0.5715	16990	0.7475	-0.0027	0.0351	0.9393	13366	0.8644	-0.0103	0.0111	0.3531	26790
rs2229742	c	g	0.1176	0.0698	0.047	0.1376	16579	0.1103	0.0045	0.0492	0.9277	12937	0.1364	-0.0044	0.0162	0.7857	26789
rs2074735	c	g	0.0689	0.0605	0.0613	0.3235	16586	0.0704	0.0704	0.0631	0.2646	12935	0.1578	0.0213	0.0149	0.1512	26789
rs960596	t	c	0.3349	-0.0088	0.0313	0.7779	16993	0.3344	0.0055	0.0319	0.8641	13373	0.3741	-0.0014	0.0099	0.885	26789
rs6438900	c	g	0.7287	0.0337	0.0329	0.3066	17017	0.7282	-0.0246	0.0339	0.4681	13385	0.748	-0.0066	0.0097	0.5	26790
rs6773343	t	c	0.7199	-0.0373	0.0323	0.2479	17037	0.7247	0.0046	0.0334	0.8901	13386	0.7932	0.0078	0.0101	0.44	26790
rs1972994	a	t	0.3415	-0.0381	0.0307	0.2146	17037	0.3446	0.0209	0.0313	0.5048	13385	0.3557	0.0018	0.0095	0.8508	26790
rs78649910	a	t	0.1197	0.0614	0.0471	0.1922	16995	0.1162	-0.0106	0.0489	0.8289	13373	0.1488	-0.0307	0.0132	0.01993	26792
rs7699711	t	g	0.493	0.0244	0.0521	0.6389	4705	0.5042	0.0991	0.0688	0.1495	2075	0.6559	-0.0022	0.0089	0.8041	26792
rs705117	t	c	0.8452	-0.0204	0.0491	0.6783	11766	0.8473	-0.0646	0.0454	0.1542	7999	0.8864	-0.0063	0.0108	0.5627	26790
rs11723621	a	g	0.7242	-0.0029	0.0329	0.9299	17031	0.7192	0.0019	0.0334	0.9556	13384	0.808	0.0015	0.0101	0.8812	26791
rs222026	a	t	0.1671	0.0826	0.0721	0.2519	4698	0.1682	0.0295	0.0966	0.7598	2075	0.2457	0.0242	0.0111	0.02888	26791
rs186881826	a	t	0.2482	-0.0156	0.0656	0.8127	4652	0.2567	0.0164	0.0885	0.8527	2064	0.3269	0.0153	0.0098	0.1201	26790
rs58073039	a	g	0.7207	-0.0093	0.0322	0.7727	17030	0.7143	0.0342	0.0332	0.3042	13386	0.7466	-0.0023	0.0095	0.8081	26791
rs7718395	c	g	0.6602	-0.0176	0.0308	0.5671	17005	0.6673	0.0323	0.0318	0.3109	13378	0.79	0.0007	0.0099	0.9435	26791
rs1011468	a	g	0.4725	0.0136	0.0292	0.6421	17023	0.4684	0.0249	0.0302	0.4102	13382	0.5819	0.0039	0.0089	0.6598	26791
rs1858889	a	c	0.5068	0.0076	0.0292	0.7947	17034	0.5063	0.0303	0.0302	0.3145	13383	0.5596	-0.0001	0.0087	0.9898	26791
rs111529171	c	g	0.2021	-0.0843	0.0366	0.02117	17022	0.2061	0.0092	0.0373	0.805	13380	0.2175	-0.0058	0.0114	0.6131	26792
rs804280	a	c	0.5946	-0.0094	0.0358	0.7938	11767	0.587	0.0001	0.0332	0.9966	7999	0.7116	-0.0033	0.0092	0.7233	26791
rs7828742	a	g	0.3991	0.0619	0.0297	0.03707	17032	0.3983	-0.0058	0.0306	0.8495	13383	0.4595	0.0182	0.0092	0.04824	26790
rs34726834	t	c	0.258	0.0278	0.0337	0.4096	17037	0.2557	0.0389	0.0346	0.2606	13385	0.3408	0.0008	0.0095	0.934	26790
rs10818769	c	g	0.1417	0.0636	0.0427	0.1364	17037	0.1419	-0.0203	0.0431	0.6374	13386	0.4206	0.0054	0.0105	0.6048	26791
rs532436	a	g	0.2008	0.0213	0.0373	0.5678	16586	0.2028	-0.0193	0.0382	0.613	12938	0.2318	-0.0021	0.0113	0.8535	26791
rs2934744	a	c	0.6596	-0.0007	0.0314	0.9824	16588	0.6568	-0.0361	0.0323	0.2644	12938	0.733	-0.0067	0.0091	0.4594	26791
rs201501563	t	c	0.5809	0.0359	0.0296	0.2263	17037	0.5812	0.0325	0.0305	0.2856	13386	0.6232	0.0175	0.0088	0.04597	26791
rs7650253	a	t	0.3065	-0.015	0.0317	0.6364	17037	0.313	0.0048	0.0325	0.8835	13386	0.4507	-0.0039	0.0098	0.6916	26789
rs3822868	g	a	0.1676	0.0219	0.0393	0.5771	17037	0.1677	-0.0472	0.0396	0.2341	13386	0.22	0.0078	0.0111	0.48	26791
rs200454003	t	c	NA	NA	NA	NA	NA	NA	NA	NA	NA	NA	0.4779	0.0127	0.0128	0.3216	12760

A1: Allele1; A2: Allele 2; A1F: Allele 1 frequency. All P values test the null hypothesis of no association between the SNP and relevant caries outcome.

### Causal effect estimation

In paediatric populations, the estimated causal effect of 25(OH)D on caries in primary and permanent teeth was essentially null ( Table 3). For caries in primary teeth the estimated effect was small in magnitude with CIs crossing the null (OR = 1.06 [95%CI: 0.81, 1.31], P = 0.66). For caries in permanent teeth the estimated effect was of no causal impact of 25(OH)D (OR = 1.00 [95%CI: 0.76, 1.23], P = 0.97).

In adult populations, the estimated causal effect of 25(OH)D was in the direction of a small protective effect, with higher 25(OH)D associated with fewer caries-affected tooth surfaces (0.31 fewer caries-affected tooth surfaces [95%CI: from 1.81 fewer DMFS to 1.19 more DMFS, P= 0.68). This effect was again small in magnitude and the CIs did not provide evidence against the null hypothesis of no effect ( Table 3).

**Table 3.** Summary of MR estimates for each dental outcome for primary inverse variance weighted analysis, weighted median sensitivity analysis and MR-Egger regression sensitivity analysis.

	Sample size (n)	Age (years)	Method	Beta	Standard error	Transformed effect	95% Confidence Intervals	P *
**Caries in primary** **teeth**	16572	3–12	Inverse Variance Weighted	0.0575	0.129	1.06	0.81, 1.31	0.66
			Weighted Median	-0.0298	0.167	0.97	0.64, 1.30	0.86
			MR-Egger	0.0187	0.178	1.01	0.67, 1.37	0.92
**Caries in** **permanent teeth**	12935	6–18	Inverse Variance Weighted	-0.00428	0.119	1.00	0.76, 1.23	0.97
			Weighted Median	0.00946	0.169	1.01	0.68, 1.34	0.96
			MR-Egger	-0.0149	0.164	0.99	0.66, 1.31	0.93
**DMFS**	26791	18–93	Inverse Variance Weighted	-0.0158	0.0385	-0.31 surfaces	-1.81, 1.19 surfaces	0.68
			Weighted Median	0.00786	0.0500	0.16 surfaces	-1.79, 2.10 surfaces	0.87
			MR-Egger	-0.0392	0.0550	-0.78 surfaces	-2.92, 1.36 surfaces	0.48

*P values test the null hypothesis of no causal association between 25(OH)D and caries outcome.

*Leave-one-out sensitivity analysis*

**Figure 1.**
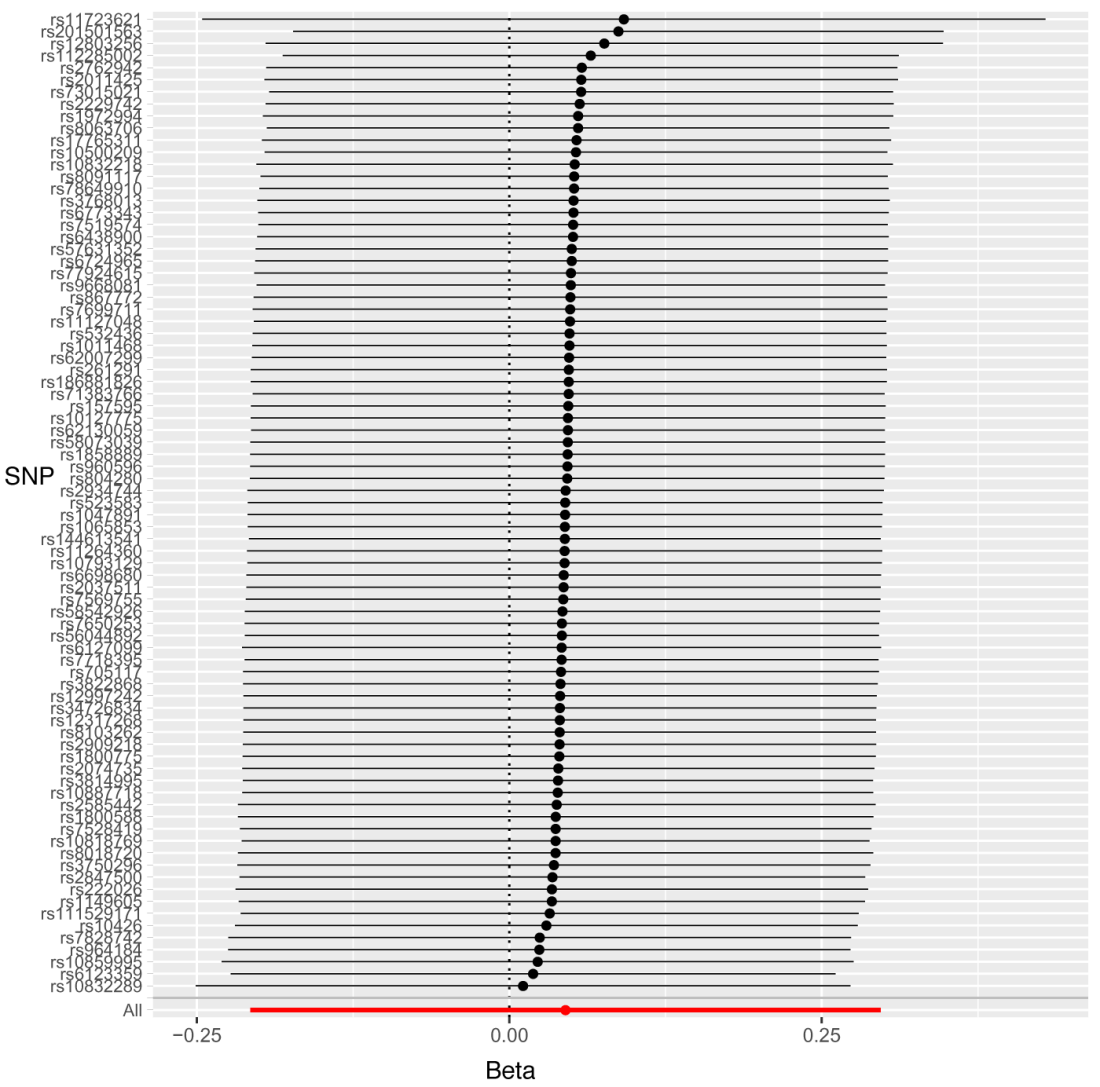
Forest plot of leave-one-out analysis of each 25(OH)D SNP on caries in primary teeth in paediatric populations.

Leave-one-out sensitivity analysis suggested that no single SNP was strongly driving the IVW point estimates for each trait, since there were no cases where excluding one SNP resulted in dramatic changes in the overall result. In addition, all the CIs overlapped with the other causal estimates in each sensitivity analysis, further suggesting that all SNPs estimated a single common exposure ( Figure 1– Figure 3).

**Figure 2.**
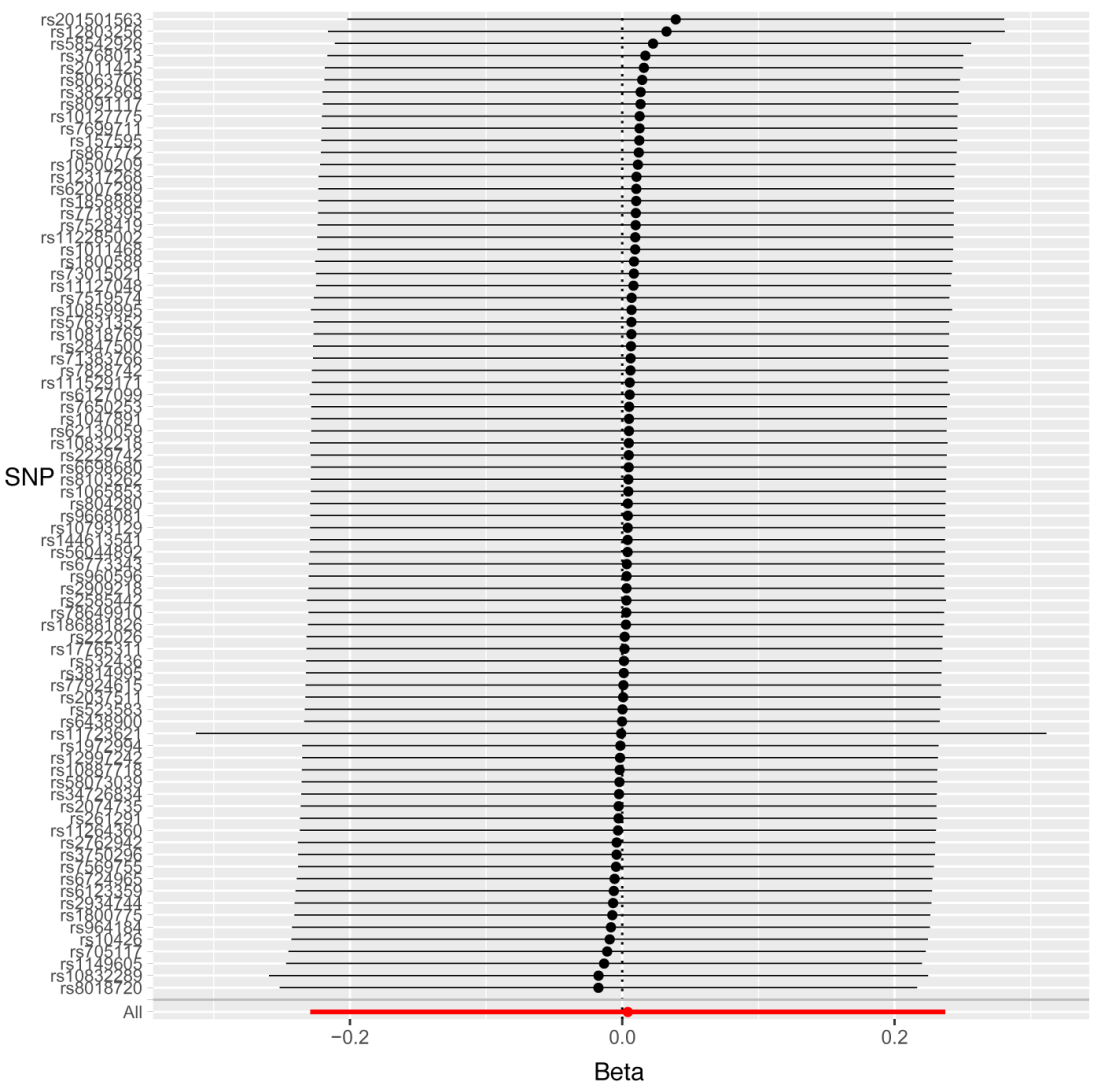
Forest plot of leave-one-out analysis of each 25(OH)D SNP on caries in permanent teeth in paediatric populations.

**Figure 3.**
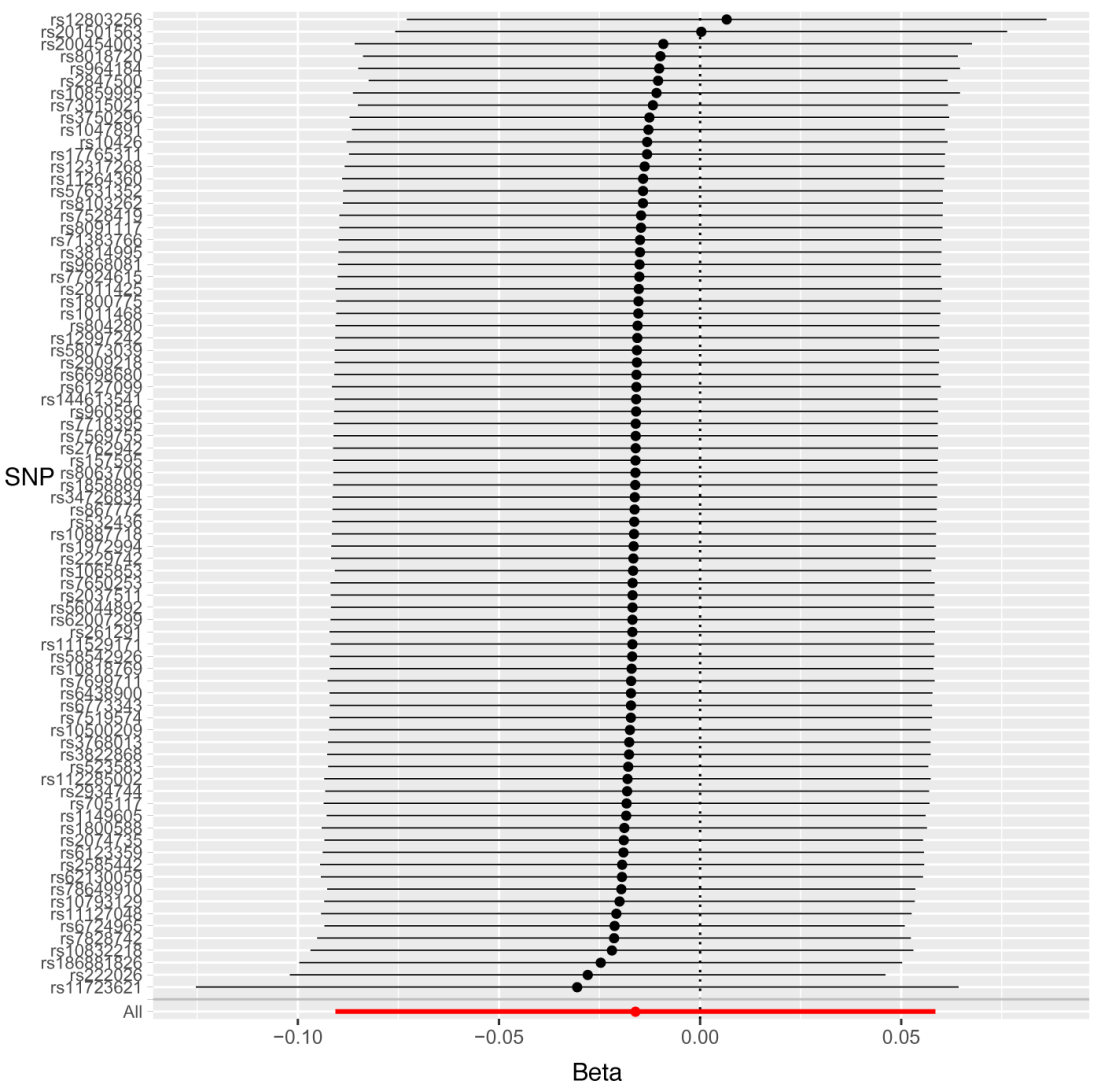
Forest plot of leave-one-out analysis of each 25(OH)D SNP on DMFS.

*Weighted median sensitivity analysis*

Weighted Median sensitivity analysis for all three traits provided effect estimates similar in magnitude to the IVW primary analysis ( Table 3). This suggests that the 25(OH)D variants were valid instruments. 

*MR-Egger sensitivity analysis*

The MR-Egger sensitivity analysis for all three traits provided effect estimates similar in magnitude to the IVW primary analysis and WM analysis ( Table 3). The MR-Egger regression analysis for all three traits found y-intercepts compatible with 0 (caries in primary teeth: y-intercept = 0.002, SE=0.006, P=0.75; caries in permanent teeth in children and adolescents: y-intercept=0.006, SE=0.006, P=0.93; DMFS: y-intercept=0.001, SE=0.002, P=0.55) and did not find evidence against the null hypothesis of no unbalanced horizontal pleiotropy.

***Phenotypic sensitivity analysis.*** Sensitivity analyses were undertaken excluding questionnaire-derived data. The sensitivity analyses included 11,344 children with data on caries in primary teeth and 7,480 children and adolescents with data on caries in permanent teeth. The effect estimates had similar interpretation to the effect estimates in primary analysis but with reduced precision (caries in primary teeth: OR=1.12 (95% CI 0.83; 1.41) P=0.45; caries in permanent teeth in children and adolescents: OR=1.11 (95% CI 0.72; 1.51) P=0.59).

***Sensitivity analysis for ancestry.*** Sensitivity analysis of DMFS, which excluded a study of Hispanic/Latino ancestry included 14,975 adults. The effect estimate was small and had reduced precision compared to the principal analysis, but agreed with the estimate from the principal analysis within the 95% CIs. The sensitivity analysis estimate was (0.43 more decayed, missing or filled tooth surfaces (95% CI -1.40; 2.27 tooth surfaces), P=0.64).

***Strength of instrumental variables.*** The 81 SNPs combined were considered to be a strong proxy for 25(OH)D in the MR study with F-statistics > 10 for all outcomes ( Burgess *et al*., 2011).

*Power*

Post-hoc power calculations suggested that the study was well-powered (β=0.8) to identify effect sizes equal to or larger than an OR = 0.8 for binary outcomes or a 1.6 surface change in DMFS per 1 SD change increase in 25(OH)D ( Table 4).

**Table 4.** Estimated detectable effect size for each trait given study power.

Outcome	Sample size (n)	Age (years)	Type-I error rate (α)	Power (β)	Proportion of cases (K)	Proportion of variance explained (R ^2^)	Effect size detectable per 1 SD increase in 25(OH)D
Caries in primary teeth	17035	3–12	0.05	0.8	0.406	0.044	OR = 0.81
Caries in permanent teeth	13386	6–18	0.05	0.8	0.439	0.044	OR = 0.79
DMFS	26792	18–93	0.05	0.8	NA	0.044	surfaces = 1.6

## Discussion

This study tested for causal effects of serum 25(OH)D on dental caries using an MR approach. The main findings are near-null causal effect estimates for all three dental caries traits which do not provide evdience against the null hypothesis of no causal association between 25(OH)D and dental caries. The effect sizes seen in this study agree with some reported associations in the observational literature, for example a weak association with a similar OR (0.97) found by Gyll *et al*. (2018) but disagrees with other studies in the literature. In particular, the causal effect sizes are smaller than the effect sizes reported by Schroth *et al*. (2016) (OR=0.57, 95% CI: 0.39, 0.82) for a comparible change in 25(OH)D.

One limitation of the MR approach is statistical power, since this method requires large samples to produce precice causal estimates. To evaluate whether the apparent disagreement in the results of this study and previous literature might be due to statistical power, we undertook post-hoc power calculations showing that with the instrument used here the study has >80% power to detect a casual association as large as those previously reported in observational studies or controlled clinical trials. We did not see large effect sizes which could be interpreted as suggesting that confounding in the observational literature and publication bias in results of controlled clinical trials has potentially over-estimated the effect sizes, however we also consider other explanations as discussed below. To exclude a subtle causal effect (OR>0.8), much larger sample sizes will be needed.

To provide valid inference, the MR method makes three assumptions. First, the genetic variants used as proxies for the exposure need to be robustly associated with the exposure of interest. This assumption has been satisfied in the current experiment since the variants were identified from published GWAS as strongly associated with 25(OH)D (P values all ≤ 5×10 ^-8^). In addition, the F-statistics are >10, therefore the IVs are considered to be strong, owing to the large sample size of available outcomes for the three traits under study ( Burgess *et al*., 2011). It therefore appears unlikely that weak instrument bias explains the lack of causal association seen in the present study. 

The second assumption states that the genetic variants must be independent of confounders of the exposure-outcome association. In general, this assumption is difficult to test in two-sample MR experiments, but violations might arise due to population stratification (different populations inheriting haplotype blocks in different frequencies due to differences in ancestries). To help address this, nearly all participants included in the MR analysis were of European ancestry and individuals are assumed to only differ with respect to the 25(OH)D loci under study. In addition, the SNP-25(OH)D SNP-caries association statistics were obtained from models which adjusted for population substructure, as described in the respective GWAS publications. There was little evidence for inflationary bias in the summary statistics of these models, suggesting that population stratification was well-controlled. Finally, sensitivity analyses were performed using more stringent exclusion criteria for ancestry in adults, and the results of these analyses agreed with the primary analysis. These approaches help protect against violations of the second assumption, but as a result of sample restriction, the results from this study are only applicable to individuals of European ancestry and are not necessarily generalizable to other ethnic populations. As a more general observation on external validity, the children included in this study were recruited from wealthy countries with longstanding public health messages regarding both vitamin D and dental caries. Thus, the prevalence of both vitamin D deficiencies and caries may be lower in the study population than in other groups, and care is needed in extrapolating the findings to populations in other countries or indeed historical studies in the same countries.

The third assumption states that the genetic instrument must be associated with dental caries, only through its effects on 25(OH)D and not via any alternative pathways (referred to as pleiotropy). If any single SNP had strongly pleiotropic effects acting on the outcome through a mechanism other than through 25(OH)D, this would be shown in the leave-one-out sensitivity analysis as an outlier. No such outliers were identified. Groups of SNPs with pleiotropic effects would result in a discrepancy between the results of IVW and WM analysis, which we did not observe. Finally, groups of SNPs with unbalanced horizontal pleiotropic effects would result in a detectable intercept term in MR-Egger regression analysis.

This experiment used a two-sample design which places natural limits on the scope of the experiment. For example, it was not possible to test for non-linear effects of 25(OH)D or describe the full characteristics of the SNP instruments. However, within these limits we have tested for we did not find evidence of violations of the assumptions of the method.

In summary, MR has provided a genetic approach to assess causality between 25(OH)D and dental caries, an association which is currently not fully understood. This study did not find evidence supporting a clinically relevant causal association. Although the assumptions required by this method appeared to be valid, results were similar across different caries outcomes and findings were consistent in sensitivity analysis, we acknowledge that statistical power was a limitation. At this moment in time the results do not suggest that vitamin D supplementation is likely to be an effective population-level risk reduction strategy for alleviating the burden of disease from dental caries where there is no suspicion of vitamin D insufficiency. In the future, larger GWAS for caries may provide more precise quantification of the role of vitamin D or other modifiable risk factors in the aetiology of this complex and important disease.

## Data availability

### Source data

University of Bristol Research Data Repository: Summary statistics of consortium GWAS for dental caries in paediatric populations. https://doi.org/10.5523/bris.pkqcnil6e9ju2nyreblt3mvwf ( Haworth & Timpson, 2018)

This project contains genome-wide summary statistics for analysis of caries traits in children. Estimates of genetic effects on caries in the primary dentition and caries in the permanent dentition in paediatric populations were extracted from this data release.

University of Bristol Research Data Repository: GWAS summary statistics for dental caries and periodontitis. https://doi.org/10.5523/bris.2j2rqgzedxlq02oqbb4vmycnc2 ( Haworth, 2019).

This project contains results of analysis in adults. Estimates of genetic effect on caries in adults (DMFS scores) were extracted from this data release.

Data are available under the Non-Commercial Government Licence for public sector information.
