# Supplementary material for: Examining the causal association between 25-hydroxyvitamin D and caries in children and adults: a two-sample Mendelian randomization approach
Source: Wellcome Open Res. 2021 Jul 20;5:281. Originally published 2020 Dec 1. [Version 2] doi: 10.12688/wellcomeopenres.16369.2 (PMC8327219; doi:10.12688/wellcomeopenres.16369.2)
Supplement: Peer review history

# Peer review: Reviewer response for version 2

**DOI:** 10.21956/wellcomeopenres.18849.r45050

**Published:** 2021-08-03

**Authors:** Yi-Qian Sun

**Affiliations:** 1 Department of Clinical and Molecular Medicine, Norwegian University of Science and Technology, Trondheim, Norway; 2 Center for Oral Health Services and Research Mid-Norway (TkMidt), Trondheim, Norway

The authors have addressed all my comments properly. I have no further comments.

Is the work clearly and accurately presented and does it cite the current literature?

Yes

If applicable, is the statistical analysis and its interpretation appropriate?

Yes

Are all the source data underlying the results available to ensure full reproducibility?

Yes

Is the study design appropriate and is the work technically sound?

Yes

Are the conclusions drawn adequately supported by the results?

Yes

Are sufficient details of methods and analysis provided to allow replication by others?

Yes

Reviewer Expertise:

Epidemiology; Mendelian randomization; vitamin D; dental health

I confirm that I have read this submission and believe that I have an appropriate level of expertise to confirm that it is of an acceptable scientific standard.

# Peer review: Reviewer response for version 2

**DOI:** 10.21956/wellcomeopenres.18849.r45051

**Published:** 2021-07-30

**Authors:** Robert J Schroth

**Affiliations:** 1 Rady Faculty of Health Sciences, University of Manitoba, Winnipeg, Canada

I am satisfied with the revisions and have no further comments.

Is the work clearly and accurately presented and does it cite the current literature?

Yes

If applicable, is the statistical analysis and its interpretation appropriate?

Yes

Are all the source data underlying the results available to ensure full reproducibility?

Yes

Is the study design appropriate and is the work technically sound?

Yes

Are the conclusions drawn adequately supported by the results?

Yes

Are sufficient details of methods and analysis provided to allow replication by others?

Yes

Reviewer Expertise:

NA

I confirm that I have read this submission and believe that I have an appropriate level of expertise to confirm that it is of an acceptable scientific standard.

# Peer review: Reviewer response for version 1

**DOI:** 10.21956/wellcomeopenres.18004.r42654

**Published:** 2021-03-11

**Authors:** Robert J Schroth

**Affiliations:** 1 Rady Faculty of Health Sciences, University of Manitoba, Winnipeg, Canada

Thank you very much for the opportunity to review this manuscript. This is a very interesting and novel study exploring whether there is evidence of a causal relationship between vitamin D and caries in children and adults. The authors have used a Mendelian Randomization (MR) approach to estimate the causal effect between serum 25(OH)D and caries.

 Title: This reviewer would suggest a change in the title so that it would be more informative to readers and indicate the type of study it is. Perhaps it would be better as “Examining the causal relationship between 25-hydroxyvitamin D and caries in children and adults: a MR analysis approach.”

 Abstract: the use of the term “null hypothesis” may not be familiar for readers from some regions of the world. It would be more clear to just state what the hypothesis was and whether the findings support the causal relationship or not.

 Introduction: is appropriate. The only comment would be that the authors have reported on findings from cross-sectional studies. Is it worth mentioning findings from any prospective studies looking at 25(OH)D during periods of tooth formation and caries at a later time point?

 Methods: perhaps the addition of an extra sentence or two about genome-wide association studies (GWASs) would help future readers. Would it be possible to add a small figure that shows the various GWASs whether data were obtained from to conduct this MR study?

 Results: well described.

 Discussion: as recommended earlier, it might be best to rephrase and avoid the use of the term “null hypothesis”.

 Tables and figures: appropriate.

Is the work clearly and accurately presented and does it cite the current literature?

Yes

If applicable, is the statistical analysis and its interpretation appropriate?

Yes

Are all the source data underlying the results available to ensure full reproducibility?

Yes

Is the study design appropriate and is the work technically sound?

Yes

Are the conclusions drawn adequately supported by the results?

Yes

Are sufficient details of methods and analysis provided to allow replication by others?

Yes

Reviewer Expertise:

Vitamin D and dental caries, early childhood caries, dentistry.

I confirm that I have read this submission and believe that I have an appropriate level of expertise to confirm that it is of an acceptable scientific standard, however I have significant reservations, as outlined above.

# Response

**Published:** 2021-07-12

**Authors:** Serena A Dodhia

**Affiliations:** University of Bristol, UK

Many thanks for taking the time to review this manuscript and for your valuable comments. We have carefully considered each comment and made changes in the paper where required. Please find point-by-point responses below. Reviewer comments are in bold type. Responses are in plain type. Sections quoted from the revised manuscript are in italics.

 1. **Title: This reviewer would suggest a change in the title so that it would be more informative to readers and indicate the type of study it is. Perhaps it would be better as “Examining the causal relationship between 25-hydroxyvitamin D and caries in children and adults: a MR analysis approach.”**

 Thank you for the comment, we have changed the title to:

*“Examining the causal association between 25-hydroxyvitamin D and caries in children and adults: a two-sample Mendelian Randomization approach.”*

**2. Abstract: the use of the term “null hypothesis” may not be familiar for readers from some regions of the world. It would be more clear to just state what the hypothesis was and whether the findings support the causal relationship or not.**

 Thank you for the suggestion, the term null hypothesis has been removed and reworded.

*“The MR-derived effect estimates for these three measures are small in magnitude with wide confidence intervals and do not provide evidence for a causal relationship between 25(OH)D and dental caries.”*

 In addition the term null hypothesis has been removed from the Results subsection MR-Egger sensitivity analyses: *“The MR-Egger regression analysis for all three traits found y-intercepts compatible with 0 (caries in primary teeth: y-intercept = 0.002, SE=0.006, P=0.75; caries in permanent teeth in children and adolescents: y-intercept=0.006, SE=0.006, P=0.93; DMFS: y-intercept=0.001, SE=0.002, P=0.55) indicating no unbalanced horizontal pleiotropy.”*

**3**. **Introduction: is appropriate. The only comment would be that the authors have reported on findings from cross-sectional studies. Is it worth mentioning findings from any prospective studies looking at 25(OH)D during periods of tooth formation and caries at a later time point?**

 Thank you for the comment. We have added information from an additional study carried out during tooth formation to the introduction. 

*“A 6-year follow up double-blind randomized clinical trial found that high-dose vitamin D supplementation during pregnancy was associated with reduced odds of enamel defects in the offspring (Nørrisgaard et al. 2019).”*

 A prospective study by Gyll *et al*. is mentioned in the discussion already.

**4. Methods: perhaps the addition of an extra sentence or two about genome-wide association studies (GWASs) would help future readers. Would it be possible to add a small figure that shows the various GWASs whether data were obtained from to conduct this MR study?**

 Thank you for the comment. A new figure (Figure 1) has been added to the methods section to further explain the two-sample MR method.

*“The data for SNP-exposure and SNP-outcomes were extracted from published GWASs. GWASs are used in genetic research to identify genetic variants that are associated with a specific trait. Figure 1 shows the steps involved in performing a two-sample MR.”*

*“Figure 1: A diagrammatic representation of the two sample mendelian randomization analysis. Instruments are identified from the exposure 25(OH)D GWAS (Manousaki et al. 2020), corresponding SNP effects are extracted from the outcome GWASs.*

*The exposure and outcome SNP effects are harmonized and then mendelian randomization analysis is performed. (Adapted from Hemani et al. 2016).”*

**5. Discussion: as recommended earlier, it might be best to rephrase and avoid the use of the term “null hypothesis”.**

 Thank you for the comment the term null hypothesis has been removed from the discussion as well.

*“The main findings are near-null causal effect estimates for all three dental caries traits which do not provide evidence to support a causal association between 25(OH)D and dental caries.”*

# Peer review: Reviewer response for version 1

**DOI:** 10.21956/wellcomeopenres.18004.r41696

**Published:** 2020-12-14

**Authors:** Yi-Qian Sun

**Affiliations:** 1 Department of Clinical and Molecular Medicine, Norwegian University of Science and Technology, Trondheim, Norway; 2 Center for Oral Health Services and Research Mid-Norway (TkMidt), Trondheim, Norway

The authors have performed a two-sample Mendelian randomization (2SMR) study to investigate the potential causal association between serum 25-hydroxyvitamin D (25(OH)D) and dental caries in children and adults. They have used publicly available summary statistics from GWASs of serum 25(OH)D and dental caries for the analyses. Little evidence was observed to support a causal effect of serum 25(OH)D on dental caries.

Major comments:

1. The answer to the research question is warranted in the dental health. The authors have used the latest GWAS summary statistics and applied primary and several robust MR methods. They have also performed sensitivity analyses excluding questionnaire-based outcome data for children and a study of Hispanic ancestry for DMFS. The conclusion and the implication of the study were based on the solid results and the three MR assumptions were addressed well in the discussion.
2. The authors used statistically driven approach to select genetic variants, i.e. included all common variants (MAF≥5%) that are associated with serum 25(OH)D at a given level of statistical significance ( *P*<6.6 x 10 ^-9^) in one of the latest GWAS for 25(OH)D ^1^. Use of many genetic variants with unclear biological functions likely results in horizontal pleiotropy of an association. In the study by Manousaki *et al* ^1^, partially shared heritability between 25(OH)D and socio-economic traits were indeed observed. The existence of pleiotropy likely changes the estimates away from the null association. However, in theory it is possible that pleiotropy leads to a false negative finding ^2^. It would be advisable if the authors could perform extra sensitivity analyses using only independent SNPs related to four loci ( *GC, NADSYN1/DHCR7, CYP2R1, CYP24A1*) ^3^ that have biological relevance to 25(OH)D to avoid horizontal pleiotropy and confirm the null associations.
3. I would like to discuss the necessity for post hoc power analysis with the authors. The power analysis that the authors applied is designed mainly for one-sample MR ^4^. As confidence intervals (CIs) indicate the precision of the observed association estimates and reflect the sample size, post hoc power analysis is generally not recommended ^5^.

Minor comments:

1. Please write clearly that the study is a two-sample MR in the title, abstract and the introduction section.

*In the Methods:*

1. One of the selection criteria for SNPs used as proxies for 25(OH)D exposure is “SNPs had independent effects”. The used linkage disequilibrium (LD) R ^2^ cut-off value should be given.
2. GWAS of DMFS: DMFS as a count variable has a strongly positively skewed distribution with a large stack of zero counts for those without caries. How the zeros were handled when DMFS scores were generated?

*In the Results*

1. The F statistic is usually reported in one-sample MR studies to represent strength of the instrumental variables (IVs). In a two-sample setting, the R ^2^ statistic (a measure of the variance in the exposure explained by the genetic variants) is more proper. In the Results, it reads “In total, these SNPs explain approximately 4.4% of the total variation in 25(OH)D levels in populations of European ancestry”. How was this 4.4% calculated? In the study by Manousaki *et al*., 138 SNPs explained 4.9% of the variation of 25(OH)D level.
2. For Table 2, could the authors rearrange the order of SNPs in the same way as in Table 1 and give chromosome positions for easier reading?
3. The tables and figures should be interpretable by themselves. Add more details in table footnotes and figure legends: e.g.
  1. Table 1. Number of participants should be given for the SNP-25(OH)D association. According to the study by Manousaki *et al*., the effect estimates and P values were based on meta-analysis (n=443,734).
  2. Table 3: use OR instead of “Transformed effect”, OR and beta correspond to genetically determined one standard deviation increase in natural log-transformed 25(OH)D.
  3. Figures 1 and 2: on risk for caries (binary); Figure 3. full name of DMFS.

Is the work clearly and accurately presented and does it cite the current literature?

Yes

If applicable, is the statistical analysis and its interpretation appropriate?

Yes

Are all the source data underlying the results available to ensure full reproducibility?

Yes

Is the study design appropriate and is the work technically sound?

Yes

Are the conclusions drawn adequately supported by the results?

Yes

Are sufficient details of methods and analysis provided to allow replication by others?

Yes

Reviewer Expertise:

Epidemiology; Mendelian randomization; vitamin D; dental health

I confirm that I have read this submission and believe that I have an appropriate level of expertise to confirm that it is of an acceptable scientific standard.

## References

[1] : Genome-wide Association Study for Vitamin D Levels Reveals 69 Independent Loci. *The American Journal of Human Genetics* .2020;106(3) : 10.1016/j.ajhg.2020.01.017 327-337 10.1016/j.ajhg.2020.01.017 PMC705882432059762

[2] : Guidelines for performing Mendelian randomization investigations. *Wellcome Open Res* .2019;4: 10.12688/wellcomeopenres.15555.2 186 10.12688/wellcomeopenres.15555.2 32760811PMC7384151

[3] : Genome-wide association study in 79,366 European-ancestry individuals informs the genetic architecture of 25-hydroxyvitamin D levels. *Nature Communications* .2018;9(1) : 10.1038/s41467-017-02662-2 10.1038/s41467-017-02662-2 PMC577264729343764

[4] : Calculating statistical power in Mendelian randomization studies. *Int J Epidemiol* .2013;42(5) : 10.1093/ije/dyt179 1497-501 10.1093/ije/dyt179 24159078PMC3807619

[5] : The Abuse of Power. *The American Statistician* .2001;55(1) : 10.1198/000313001300339897 19-24 10.1198/000313001300339897

# Response

**Published:** 2021-07-12

**Authors:** Serena A Dodhia

**Affiliations:** University of Bristol, UK

Thank you for taking the time to review this submission and for the valuable comments. We have considered each comment and made changes in the paper where required. Please find point-by-point responses below. Reviewer comments are in bold type. Responses are in plain type. Sections quoted from the revised manuscript are in italics.

**1. The authors used statistically driven approach to select genetic variants, i.e. included all common variants (MAF≥5%) that are associated with serum 25(OH)D at a given level of statistical significance ( *P*<6.6 x 10 ^-9^) in one of the latest GWAS for 25(OH)D. Use of many genetic variants with unclear biological functions likely results in horizontal pleiotropy of an association. In the study by Manousaki *et al*, partially shared heritability between 25(OH)D and socio-economic traits were indeed observed. The existence of pleiotropy likely changes the estimates away from the null association. However, in theory it is possible that pleiotropy leads to a false negative finding. It would be advisable if the authors could perform extra sensitivity analyses using only independent SNPs related to four loci ( *GC, NADSYN1/DHCR7, CYP2R1, CYP24A1*) that have biological relevance to 25(OH)D to avoid horizontal pleiotropy and confirm the null associations.**

 Thank you for this comment. As suggested we have included additional sensitivity analysis using a subset of variants (rs705117, rs222026, rs201501563, rs186881826, rs11723621, rs10832289 and rs10832218). These are located in the GC and CYP2R1 loci. These variants were selected as they were available in the outcome data and they had a minor allele frequency of 0.05 or above as stated in the SNP-exposure methods. The results of this analysis were concordant with the main analysis and had similar interpretation. We have added a paragraph to the results section:

*“ Sensitivity analysis using 7 independent SNPS*

*Sensitivity analyses using 7 independent SNPs, located in the the GC and CYP2R1 loci, that have biological relevance to 25(OH)D was carried out, to avoid horizontal pleiotropy and confirm the null associations. Results of this sensitivity analysis were consistent with the principal analysis (caries in primary teeth OR= 1.00 (95% CI 0.74; 1.33); caries in permanent teeth OR= 1.02 (95% CI 0.75; 1.37); DMFS 0.52 surfaces (95% CI -2.37; 3.41 surfaces).”*

**2. I would like to discuss the necessity for post hoc power analysis with the authors. The power analysis that the authors applied is designed mainly for one-sample MR** **. As confidence intervals (CIs) indicate the precision of the observed association estimates and reflect the sample size, post hoc power analysis is generally not recommended** **.**

 Thank you for this comment. We agree that the power analysis applied is redundant given the reported confidence intervals and have therefore removed Table 4 and this analysis from the methods, results and discussion. The discussion has been reworded to describe the wide confidence intervals as an indication of the greater sample size that is needed to provide more precise effect estimates.

*“The apparent disagreement in the results of this study and previous literature might be due to statistical power, the wide 95% CIs for all three caries outcome traits reflect the need for much larger sample sizes. We did not see large effect sizes which could be interpreted as suggesting that confounding in the observational literature and publication bias in results of controlled clinical trials has potentially over-estimated the effect sizes, however we also consider other explanations as discussed below.”*

**3. Please write clearly that the study is a two-sample MR in the title, abstract and the introduction section.**

 Thank you for this comment. This description has been added to the title, abstract and introduction section.

**4. One of the selection criteria for SNPs used as proxies for 25(OH)D exposure is “SNPs had independent effects”. The used linkage disequilibrium (LD) R ^2^ cut-off value should be given.**

 Thank you for this comment. The SNPs were chosen based on a conditional analysis implemented in GCTA, as reported in the original manuscript. Variants were selected if they reached genome-wide significance conditional on the lead variant in the same locus. Variants situated more than 20,000 base pairs away and variants with LD R ^2^>0.9 were excluded. For further details of GCTA-COJO analysis please refer to the section "Approximate Conditional Association Analysis" in the following paper titled “Genome-wide Association Study for Vitamin D Levels Reveals 69 Independent Loci”: https://pubmed.ncbi.nlm.nih.gov/32059762/.

*“c) were conditionally independent of other SNPs in the same genomic locus in conditional analysis *(Manousaki et al., 2020) *.”*

**5. GWAS of DMFS: DMFS as a count variable has a strongly positively skewed distribution with a large stack of zero counts for those without caries. How the zeros were handled when DMFS scores were generated?**

 Thank you for this comment. The GWAS for DMFS was carried out in adult populations. DMFS scores were regressed on age, age squared, and study specific-covariates and the residuals were plotted. We did observe skewed distributions for other traits (for example number of teeth), but not for DMFS, where the residuals were approximately normally distributed. This may be because the study population included middle aged and older adults with high disease burden. DMFS residuals were subsequently treated as a continuous trait in GWAS analysis.

**6.** **The F statistic is usually reported in one-sample MR studies to represent strength of the instrumental variables (IVs). In a two-sample setting, the R ^2^ statistic (a measure of the variance in the exposure explained by the genetic variants) is more proper. In the Results, it reads “In total, these SNPs explain approximately 4.4% of the total variation in 25(OH)D levels in populations of European ancestry”. How was this 4.4% calculated? In the study by Manousaki *et al*., 138 SNPs explained 4.9% of the variation of 25(OH)D level.**

 Thank you for this comment. While we aimed to provide some information on the potential strength of the MR instrument, we accept that the F statistic is potentially confusing and have therefore removed this part. We have amended the Results section:

*“ Strength of Instrumental Variables*

*The 81 SNPs combined were considered to be a strong proxy for 25(OH)D in the MR study. The SNPs explain approximately 4.4% of the total variation in 25(OH)D levels in populations of European ancestry.”*

 The R ^2 ^of the 25(OH)D reported in the results was estimated as the sum of variance explained by each SNP in the experiment. The variance of each SNP was given using the formula provided by the authors of the Vitamin D GWAS paper; variance explained ≈2β ^2^ ƒ(1– ƒ), where β and ƒ denote the effect estimate and the effect allele frequency of the allele on a standardized phenotype, respectively. This information has been added to the methods section.

*“The total variance explained in 25(OH)D by the SNPs was estimated as the sum of the variance explained by each SNP in the experiment. The variance of each SNP was given using the formula provided by the authors of the 25(OH)D GWAS paper: variance explained ≈ 2β ^2^ ƒ(1– ƒ), where β and ƒ denote the effect estimate and the effect allele frequency of the allele on a standardized phenotype, respectively (Manousaki et al., 2020).”*

**7. For Table 2, could the authors rearrange the order of SNPs in the same way as in Table 1 and give chromosome positions for easier reading?**

 Thank you for the suggestion, Table 2 has been rearranged to the same way as Table 1.

**8.** **The tables and figures should be interpretable by themselves. Add more details in table footnotes and figure legends: e.g.Table 1. Number of participants should be given for the SNP-25(OH)D association. According to the study by Manousaki *et al*., the effect estimates and P values were based on meta-analysis (n=443,734).**

 Thank you for the comment. More details have been added to the footnotes and figure legends.

**9.** **Table 3: use OR instead of “Transformed effect”, OR and beta correspond to genetically determined one standard deviation increase in natural log-transformed 25(OH)D.**

 Thank you for the comment. The DMFS effect estimate is not on a odds ratio scale and therefore we prefer to use the term ‘transformed effect’ for this variable. The column has been renamed to “Odds ratio/transformed effect”.

**10. Figures 1 and 2: on risk for caries (binary); Figure 3. full name of DMFS.**

 Thank you for this comment. We have added the information as suggested.
